# Interpretable Machine Learning Analysis of Design Factors in Hydrogel Supercapacitors

**DOI:** 10.3390/gels11060464

**Published:** 2025-06-18

**Authors:** Liying Xu, Siqi Liu, Dandan Hu, Junhao Liu, Yuze Zhang, Ziqiang Li, Zichuan Su, Daxin Liang

**Affiliations:** 1 School of Food Engineering, Harbin University, Harbin 150086, China; 2 Key Laboratory of Bio-Based Material Science and Technology (Ministry of Education), Northeast Forestry University, Harbin 150040, China; siqiliu61@163.com (S.L.); 17868651902@nefu.edu.cn (D.H.); 2022210600@nefu.edu.cn (J.L.); zyz050611@163.com (Y.Z.); lzqqqq2001@163.com (Z.L.); 3 College of Chemistry and Chemical Engineering, China University of Petroleum (East China), Qingdao 266580, China; z22060089@s.upc.edu.cn

**Keywords:** hydrogel supercapacitor, machine learning, interpretable prediction

## Abstract

Understanding the relationships between design factors is crucial for the development of hydrogel supercapacitors, yet the relative importance and interdependencies of material properties and operating conditions remain unclear. This study employs interpretable machine learning to analyze the design factors that affect hydrogel supercapacitor performance, using 232 experimental samples from 41 recent studies. SHAP analysis was implemented to quantify parameter importance and reveal feature interactions among 16 key design parameters, including polymer types, electrolyte formulations, and operating conditions. Results show that synthetic vinyl polymers most strongly influence specific capacitance, while conductive polymers predominantly affect cycle stability. Ionic conductivity emerged as the most impactful parameter despite moderate feature importance, indicating complex nonlinear relationships. Critical interdependencies between polymer concentration and electrolyte formulation suggest that optimal design requires coordinated parameter selection rather than independent optimization. This interpretable framework provides quantitative insights into design factor hierarchies and parameter interdependencies, offering evidence-based guidelines for rational material selection in hydrogel supercapacitor development.

## 1. Introduction

Supercapacitors have emerged as critical energy storage devices that bridge the gap between conventional capacitors and batteries, offering unique advantages of high power density, rapid charge–discharge capabilities, and exceptional cycle stability [1]. With the growing demand for sustainable energy solutions and the proliferation of portable electronics, electric vehicles, and grid-scale energy storage systems, supercapacitors have found extensive applications ranging from consumer electronics and automotive systems to renewable energy integration and emergency power backup [2]. Their ability to deliver high power output within seconds while maintaining long-term reliability makes them indispensable components in modern energy infrastructure, particularly in applications requiring quick energy bursts and frequent cycling operations [3].

Hydrogel-based supercapacitors have attracted significant attention as promising candidates for flexible energy storage applications due to their unique combination of high ionic conductivity, mechanical flexibility, and environmental adaptability [4,5,6]. Recent studies have demonstrated that hydrogel electrolytes can achieve exceptional ionic conductivity owing to their three-dimensional polymer networks with high water content, facilitating efficient ion transport and enhanced electrochemical performance [7,8]. Li et al. developed flexible supercapacitors based on organohydrogel electrolytes that exhibit remarkable long-term anti-freezing and anti-drying properties, addressing key challenges in practical applications [9]. The structural design of hydrogel electrodes has also proven crucial for performance optimization, as evidenced by Huang et al., who employed multi-scale structural engineering strategies to develop high-performance MXene hydrogel supercapacitor electrodes with enhanced capacitive behavior [10]. The self-healing capability of hydrogel electrolytes represents another significant advantage, as reported by Zhao et al., who developed self-healing hydrogel electrolytes for flexible solid-state supercapacitors, enabling device recovery from mechanical damage and extending operational lifespan [11]. These inherent properties of hydrogels, including tunable mechanical strength, biocompatibility, and environmental stability, make them superior alternatives to conventional rigid electrolyte systems for next-generation flexible and wearable energy storage devices.

However, the development of high-performance hydrogel-based supercapacitors faces significant challenges in optimizing the complex interplay between polymer composition, electrolyte formulation, and operating conditions [12,13,14,15]. Traditional trial-and-error approaches for material design are time-consuming, resource-intensive [16], and often fail to capture the intricate relationships between multiple design parameters and performance outcomes [17]. The vast parameter space involving polymer types, electrolyte concentrations, ionic conductivity, current density, and voltage windows creates a multidimensional optimization problem that is difficult to navigate using conventional experimental methods [7,18,19]. However, interpretable machine learning (ML) approaches offer unprecedented opportunities to understand material design principles by identifying complex relationships in experimental data, quantifying parameter importance, and revealing the underlying factors governing optimal material combinations [20,21].

**Figure 1:**
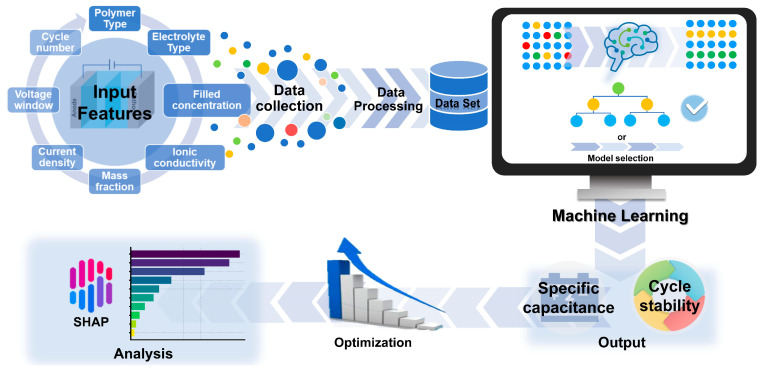
Workflow of interpretable machine learning framework for hydrogel supercapacitor performance prediction.

In this work, we developed a comprehensive interpretable machine learning framework to understand the design factors governing hydrogel-based supercapacitor performance and elucidate their relative importance. A systematic literature survey was conducted to collect 232 experimental data samples encompassing diverse material compositions and operating conditions to analyze parameter influences and material dependencies [9,10,11,12,13,15,22,23,24,25,26,27,28,29,30,31,32,33,34,35,36,37,38,39,40,41,42,43,44,45,46,47,48,49,50,51,52,53,54,55,56]. The dataset includes key parameters such as polymer types, electrolyte formulations, ionic conductivity, current density, voltage windows, and cycling conditions as input features to analyze their relationships with specific capacitance and cycle stability. Multiple machine learning algorithms were systematically evaluated to model these complex relationships, with SHAP analysis employed to quantify parameter importance and reveal the differential impacts of various polymer categories and material parameters on supercapacitor performance [57]. This interpretable approach provides mechanistic insights into design factor hierarchies and parameter interdependencies, offering evidence-based guidelines for understanding the critical factors governing hydrogel supercapacitor performance and rational material selection strategies. The overall workflow of this study is illustrated in Figure 1.

## 2. Results and Discussion

### 2.1. Analysis of Input Features Prior to Model Development

To understand the interrelationships among input parameters [17,58], Pearson correlation analysis was performed on all features before model development. The input parameters include: Polymer Concentration (PC), Alkali Metal (AM), Acid System (AS), Zinc System (ZS), Mixed System (MS), Organic Additive (OA), electrolyte Mass Fraction (MF), Ionic Conductivity (IC), Current Density (CD), Voltage Window (VW), Cycle Number (CN), Synthetic Vinyl (SV), Natural Polysaccharide (NP), Conductive Polymer (CP), Polybase (PB), and Polyacid (PA).

**Figure 2:**
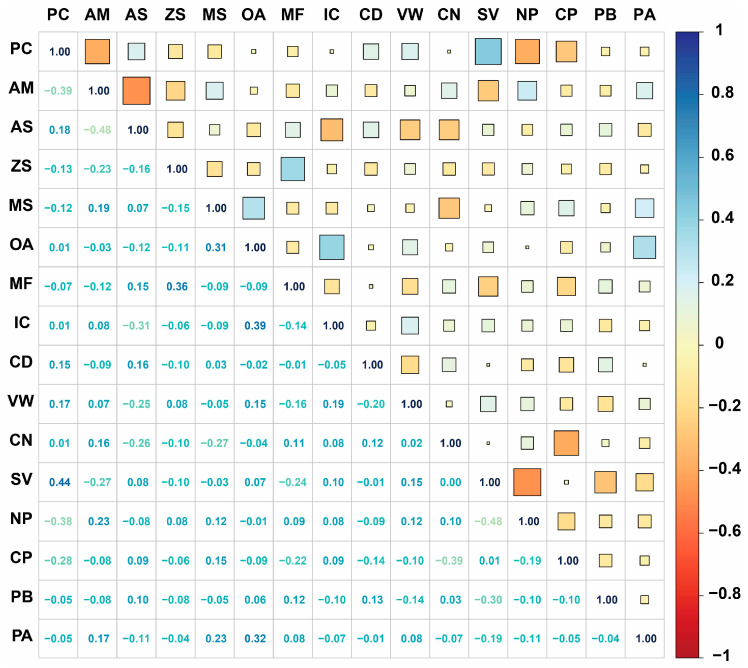
Pearson correlation matrix of input features.

The correlation matrix in Figure 2 reveals generally weak to moderate correlations among most input features, with the majority of correlation coefficients ranging from −0.5 to 0.5. This indicates that the selected features provide relatively independent information for model training, which is beneficial for machine learning performance. Notable observations include moderate positive correlations between certain electrolyte system parameters (OA-IC: 0.39, MS-OA: 0.31) and some polymer type relationships. The electrolyte MF shows weak correlations with most other parameters, suggesting its independent contribution to supercapacitor performance [24,27,31,42].

Among polymer type features, some expected relationships are observed, such as the moderate negative correlation between SV and NP (−0.48), reflecting their distinct chemical nature. The relatively low correlation values across most feature pairs indicate minimal multicollinearity issues, supporting the robustness of subsequent machine learning model development. This correlation analysis confirms that the feature engineering approach effectively captures diverse and complementary aspects of hydrogel supercapacitor design parameters [2,59,60].

Among polymer type features, the most significant correlation is the negative relationship between SV and NP at −0.48, which reflects their fundamentally different chemical origins and application strategies. The SV category encompasses synthetic polymers including polyvinyl alcohol (PVA), polyacrylamide (PAM), and polyacrylonitrile (PAN), which are characterized by controlled molecular structures, predictable crosslinking behaviors, and consistent mechanical properties. In contrast, the NP category includes natural biopolymers such as sodium alginate (SA), cellulose, chitosan (CS), alginate, and starch, which offer excellent biocompatibility, strong crosslinking capabilities through inherent functional groups, and sustainable material advantages [41,43,46,49]. The Conductive Polymer (CP) category, comprising polyaniline (PANI), polypyrrole (PPY), polyethylenedioxythiophene (PEDOT), and polythiophene (PTH), shows relatively weak correlations with other polymer types, indicating its specialized role in enhancing electrical conductivity rather than serving as a primary structural matrix. Similarly, protein-based (PB) polymers, including gelatin, collagen, albumin, and lignin, demonstrate independent behavior, reflecting their unique position in biocompatible and sustainable supercapacitor designs [51,52,53]. This correlation pattern suggests that material selection follows targeted performance criteria: synthetic vinyl polymers for mechanical reliability, natural polysaccharides for biocompatibility and flexibility, conductive polymers for electrical enhancement, and protein-based materials for specialized biomedical applications.

### 2.2. Model Development

**Figure 3:**
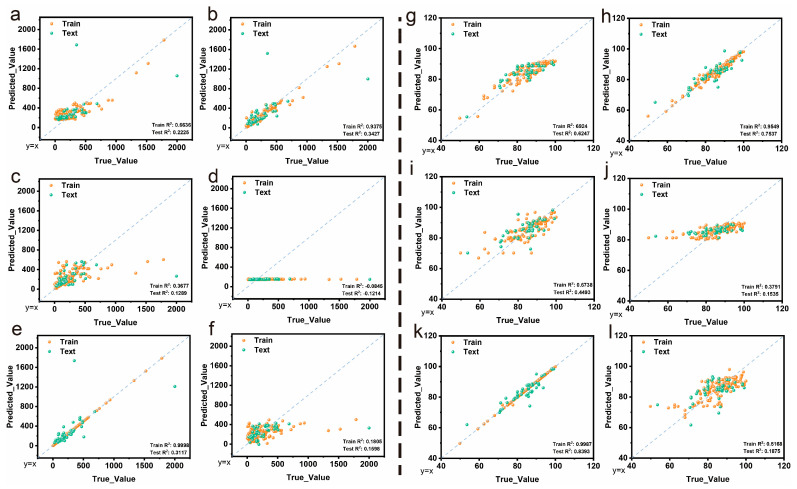
Machine learning model performance for specific capacitance (**a**–**f**) and cycle stability (**g**–**l**). (**a**,**g**) AdaBoost, (**b**,**h**) GBR, (**c**,**i**) KNN, (**d**,**j**) SVM, (**e**,**k**) XGBoost, (**f**,**l**) Lasso.

Six machine learning algorithms were systematically evaluated for predicting both specific capacitance and cycle stability of hydrogel supercapacitors. The performance of each model was assessed using training and testing datasets, with results presented as predicted vs. true value plots in Figure 3.

For specific capacitance prediction (Figure 3a–f), all models demonstrated poor overall performance with testing R^2^ values below 0.35. Notably, XGBoost (Figure 3e) exhibited significant overfitting with an extremely high training R^2^ of 0.9996 but a testing R^2^ of only 0.3117, suggesting that while XGBoost has the potential to capture the underlying patterns in specific capacitance data, proper hyperparameter optimization is crucial to improve generalization performance. The other algorithms showed consistently poor performance across both training and testing datasets, with SVM (Figure 3d) particularly struggling to fit the data [61].

For cycle stability prediction (Figure 3g–l), the models generally achieved superior performance compared to specific capacitance prediction. GBR (Figure 3h) demonstrated exceptional performance with training R^2^ of 0.9549 and testing R^2^ of 0.7537, indicating both high accuracy and excellent generalization capability. XGBoost (Figure 3k) also showed strong performance with training R^2^ of 0.9897 and testing R^2^ of 0.3393. AdaBoost (Figure 3g), KNN (Figure 3i), and Lasso (Figure 3l) achieved reasonable performance with testing R^2^ values ranging from 0.1675 to 0.6483. Notably, SVM (Figure 3j) again showed poor fitting with training R^2^ of 0.3791 and testing R^2^ of 0.1535.

The superior performance of tree-based ensemble methods (GBR and XGBoost) suggests that the relationships between input features and target properties involve complex nonlinear interactions that are effectively captured by these algorithms. The systematic model comparison through Taylor diagram analysis (Figure S1) confirmed the superior performance of optimized XGBoost for specific capacitance prediction and GBR for cycle stability prediction, while violin plot analysis (Figure S2) revealed distinct error distribution patterns that provide insights into model reliability and prediction consistency. The consistently poor performance of SVM indicates that the dataset characteristics may not be suitable for this particular algorithm’s assumptions [62].

### 2.3. Hyperparameter Optimization

**Figure 4:**
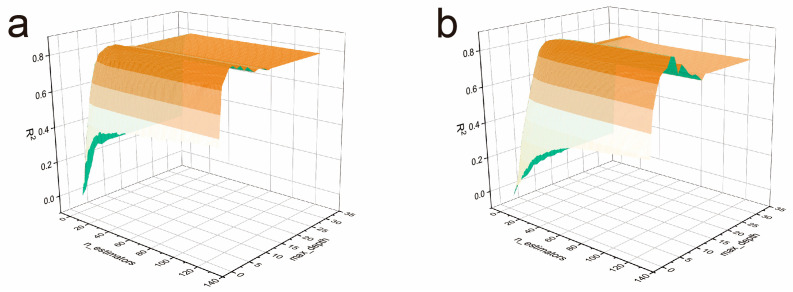
(**a**) XGBoost optimization for specific capacitance prediction and (**b**) GBR optimization for cycle stability prediction.

To address the overfitting issues identified in the initial model evaluation and improve predictive performance, systematic hyperparameter optimization was conducted for the most promising algorithms. The cross-validation results demonstrate that XGBoost optimization for cycle stability prediction achieved R^2^ = 0.857 and GBR optimization for specific capacitance prediction achieved R^2^ = 0.821, which are lower than the optimal pairings shown in Figure 4. These comparative results validate our target-specific model selection approach, confirming that XGBoost is most suitable for specific capacitance prediction while GBR excels in cycle stability prediction.

Figure 4a illustrates the hyperparameter optimization landscape for XGBoost in specific capacitance prediction, revealing a complex three-dimensional performance surface with multiple local optima and valleys. The optimization trajectory demonstrates the systematic exploration of the parameter space, starting from relatively low R^2^ values and gradually ascending toward optimal regions. The surface topology indicates that XGBoost performance is highly sensitive to hyperparameter selection, with dramatic variations in R^2^ across different parameter combinations. Notable features include steep gradients in certain regions, suggesting that small parameter changes can lead to significant performance differences [62,63].

Similarly, Figure 4b presents the hyperparameter optimization results for GBR in cycle stability prediction. The optimization landscape shows a smoother performance surface with a well-defined optimal region, indicating more stable behavior across different parameter combinations. The GBR optimization successfully identified parameter settings that maintain the model’s strong fitting capability while enhancing generalization performance, building upon the already promising initial results for cycle stability prediction.

### 2.4. Feature Importance and Interpretability Analysis

**Figure 5:**
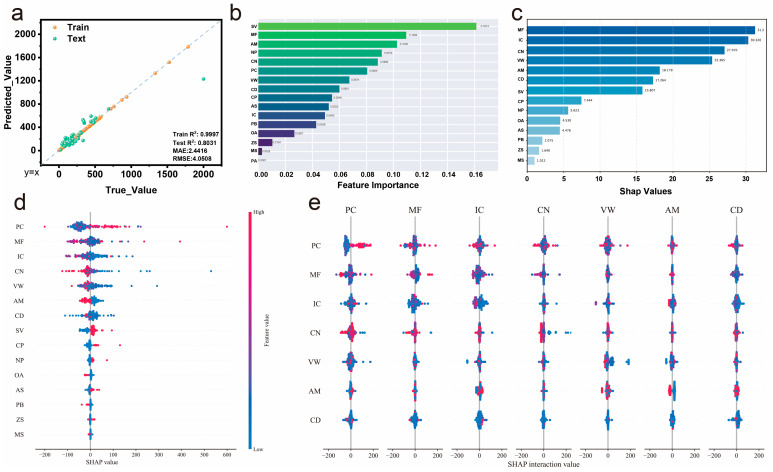
XGBoost analysis for specific capacitance prediction. (**a**) Optimized model performance, (**b**) feature importance ranking, (**c**) SHAP value summary, (**d**) SHAP value distribution for individual features, and (**e**) SHAP interaction analysis showing feature interdependencies.

The optimized XGBoost model for specific capacitance prediction shows substantially improved performance compared to the initial model (Figure 5a). The predicted vs. true value plot reveals excellent fitting with a training R^2^ of 0.99 and testing R^2^ of 0.8, representing a significant improvement in generalization capability while maintaining high training accuracy. The mean absolute error (MAE) of 2.44 and root mean square error (RMSE) of 4.05 indicate reasonable prediction precision across the range of specific capacitance values. The well-distributed data points along the diagonal line demonstrate that the hyperparameter optimization successfully addressed the overfitting issues identified in the preliminary analysis. Feature importance analysis (Figure 5b) reveals the relative contribution of different input parameters to specific capacitance prediction. SV emerges as the most influential feature with an importance score of approximately 0.15, suggesting that synthetic vinyl polymers like PVA, PAM, and PAN play a crucial role in determining capacitive performance. MF ranks as the second most important feature, indicating that electrolyte concentration significantly affects specific capacitance [34,37]. NP and CP also show substantial importance, reflecting the significant impact of polymer type selection on capacitive behavior [24,45,48]. Notably, CN and PC demonstrate moderate importance with feature importance scores of approximately 0.10 and 0.08, respectively, indicating that cycling protocols and polymer concentration have secondary but meaningful impacts on specific capacitance. The moderate influence of PC reflects the critical balance between mechanical integrity and ionic accessibility—optimal polymer concentrations must maintain structural stability while avoiding impedance of ion transport pathways [32,34]. In contrast, electrolyte system features (ZS, MS, PA) show relatively lower influence (importance scores < 0.06), suggesting that physical properties such as ionic conductivity and mass fraction are more critical than specific electrolyte chemistry for capacitive performance [39,41,43].

SHAP summary analysis (Figure 5c) provides deeper insights into feature contributions by quantifying the impact magnitude of each parameter. IC shows the highest SHAP values at approximately 30, indicating its dominant influence on model predictions despite its moderate feature importance ranking. This apparent discrepancy suggests that while IC may not be frequently used for decision-making in the tree structure, its impact when activated is substantial. CN and PC display significant SHAP values around 27 and 24, respectively, confirming their important roles in specific capacitance determination [37,49]. The detailed SHAP value distribution (Figure 5d) illustrates how individual features contribute positively or negatively to predictions across different samples. PC, MF, IC, and CN exhibit the widest SHAP value ranges, with PC showing both substantial positive and negative contributions, indicating highly context-dependent effects on specific capacitance. PC exhibits the widest SHAP range, with both positive and negative contributions evenly distributed, indicating that polymer concentration effects are highly context-dependent and can either enhance or inhibit specific capacitance based on other system parameters [11,25]. MF shows a similarly broad range but with a slight bias toward positive contributions, suggesting that while electrolyte mass fraction generally benefits capacitive performance, optimal values depend on polymer matrix compatibility [55,56,62]. IC demonstrates predominantly positive SHAP values in the high-impact region, confirming its consistently beneficial role, while the color gradient shows that higher ionic conductivity values (red dots) correlate with stronger positive contributions [52,53]. Mid-range features, including VW, AM, CD, and SV, display moderate SHAP distributions, with SV showing primarily positive contributions that support its beneficial effect on capacitive performance. The remaining features demonstrate narrow SHAP distributions concentrated near zero, indicating stable but limited contributions to specific capacitance prediction. This hierarchical pattern confirms our feature importance ranking while revealing that high-impact features exhibit greater variability in their effects, whereas low-importance features provide consistent but modest contributions.

SHAP interaction analysis (Figure 5e) reveals the complex interdependencies between different input features. The interaction heatmap shows that PC exhibits significant interactions with multiple other features, particularly with MF and IC. These interactions suggest that the optimal polymer concentration depends on electrolyte formulation and conductivity levels, highlighting the importance of synergistic material design [11,12,23]. These interaction patterns provide valuable guidance for material optimization, suggesting that holistic design approaches considering parameter interdependencies are essential for achieving optimal specific capacitance performance.

**Figure 6:**
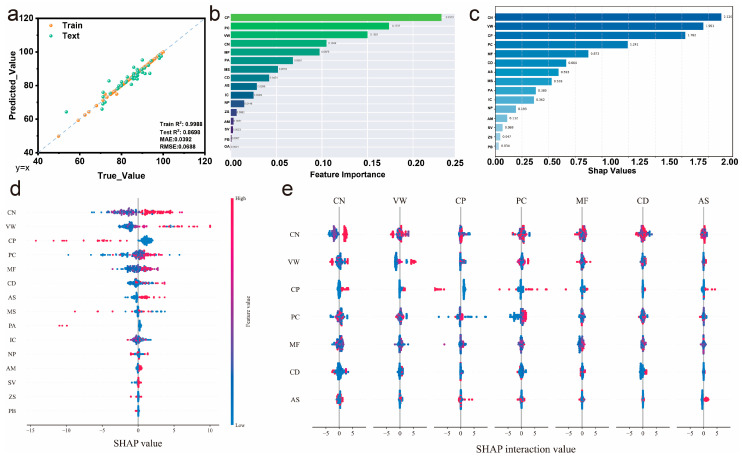
GBR analysis for cycle stability. (**a**) Optimized model performance, (**b**) feature importance ranking, (**c**) SHAP value summary, (**d**) SHAP value distribution for individual features, and (**e**) SHAP interaction analysis showing feature interdependencies.

The optimized GBR model achieves outstanding predictive performance for cycle stability (Figure 6a), with training R^2^ of 0.9988 and testing R^2^ of 0.8698, demonstrating excellent generalization capability that surpasses the specific capacitance prediction results. The MAE of 0.0392 and RMSE of 0.0688 indicate high prediction precision across the cycle stability range. Feature importance analysis (Figure 6b) reveals distinct patterns compared to specific capacitance prediction. CP emerges as the most critical feature with an importance score of approximately 0.23, indicating that conductive polymers play a dominant role in determining cycle stability. This finding suggests that the inherent electrical conductivity and structural stability of conductive polymers are crucial for maintaining performance during extended cycling. PC ranks second in importance, followed by VW and CN, indicating that polymer concentration, voltage window selection, and cycling protocols significantly influence long-term stability. Notably, MF shows moderate importance, while electrolyte system features demonstrate relatively lower influence on cycle stability.

SHAP summary analysis (Figure 6c) quantifies the impact magnitude of each feature on cycle stability predictions. CN shows the highest SHAP values at approximately 1.95, reflecting its fundamental role in defining cycling performance metrics. CP displays substantial SHAP values around 1.79, confirming its critical importance identified in the feature importance analysis [24,29,31]. MF, CD, and AS show moderate SHAP values, indicating their secondary but meaningful contributions to cycle stability. The relatively concentrated SHAP value distribution compared to specific capacitance analysis suggests more predictable and consistent feature impacts on cycle stability. The detailed SHAP value distribution (Figure 6d) illustrates the directional impacts of individual features on cycle stability predictions. CN and VW show the widest SHAP value ranges, indicating high variability in their contributions depending on specific experimental conditions. CP exhibits predominantly positive contributions, confirming that conductive polymer incorporation generally enhances cycle stability. PC shows balanced positive and negative contributions, suggesting that an optimal polymer concentration exists and deviations in either direction can negatively impact stability. The narrower SHAP distributions for electrolyte system features indicate more consistent and predictable impacts on cycle stability [37,38].

SHAP interaction analysis (Figure 6e) reveals critical interdependencies between features affecting cycle stability. CN shows strong interactions with multiple parameters, particularly with VW, CP, and PC, indicating that optimal cycling protocols must consider material composition and operating conditions. The significant interaction between VW and CP suggests that voltage window selection should be tailored based on conductive polymer content to maximize cycle stability [15,40,42]. PC exhibits notable interactions with MF and CD, implying that polymer concentration optimization requires consideration of electrolyte formulation and current density conditions. These interaction patterns highlight the importance of integrated design approaches for achieving superior cycle stability, where material selection, concentration optimization, and operating parameter selection must be coordinated to prevent premature degradation and maintain long-term performance [46,47].

## 3. Conclusions

This study successfully developed an interpretable machine learning framework for understanding design factors in hydrogel-based supercapacitors using 232 experimental samples from the literature. The systematic evaluation of six ML algorithms enabled a comprehensive analysis of parameter–performance relationships, with hyperparameter optimization ensuring robust model behavior for reliable feature importance assessment. SHAP analysis provided quantitative insights into the relative importance of design factors and their complex interdependencies.

SHAP interpretability analysis revealed key insights into design factor hierarchies and material dependencies. For specific capacitance, synthetic vinyl polymers (SV) emerged as the most influential material category, while ionic conductivity (IC) demonstrated the highest impact magnitude, emphasizing its critical role in capacitive performance. For cycle stability, conductive polymers (CP) proved most important, highlighting how PANI, PPY, PEDOT, and PTH significantly influence long-term performance maintenance [64]. SHAP interaction analysis identified crucial parameter interdependencies, particularly between polymer concentration (PC) and mass fraction (MF), revealing that coordinated parameter selection is essential for understanding optimal design strategies. The correlation analysis validated the feature engineering approach, with weak to moderate correlations ensuring minimal multicollinearity and enabling reliable importance assessment. The distinct material categories demonstrated appropriate independence, facilitating effective analysis of different polymer contributions to electrochemical performance.

This framework provides valuable insights for understanding hydrogel supercapacitor design principles by revealing parameter importance hierarchies and material dependencies through systematic analysis of literature data. The interpretable machine learning approach successfully identified critical design factors and their interdependencies. The comprehensive analysis offers evidence-based guidelines for rational material selection and demonstrates the potential of interpretable AI in elucidating complex material–property relationships.

However, a major challenge encountered during data collection was the lack of standardization in experimental protocols and reporting formats across different studies. Variations in testing conditions, measurement methods, and data presentation significantly hindered comprehensive analysis and limited the development of more sophisticated machine learning models. This heterogeneity represents a critical bottleneck for advancing data-driven materials research in the hydrogel supercapacitor field. To unlock the full potential of machine learning in materials discovery, we advocate for the research community to establish standardized experimental protocols, unified data reporting formats, and open-access databases with consistent metadata. Such initiatives would enable more robust predictive models, facilitate deeper mechanistic understanding, and accelerate the transition toward systematic data-driven materials design, ultimately realizing the transformative potential of artificial intelligence in hydrogel-based energy storage technologies [65,66].

## 4. Materials and Methods

### 4.1. Data Collection and Preprocessing

This study utilized Web of Science as the primary data source, employing “hydrogel supercapacitor”, “hydrogel electrolyte”, and “flexible supercapacitor” as search keywords to systematically collect experimental data from peer-reviewed literature published between 2020 and 2024. The literature survey employed systematic screening criteria including: (1) experimental studies with complete electrochemical performance data, (2) clear polymer and electrolyte specifications, and (3) quantitative reporting of both specific capacitance and cycle stability, resulting in 41 relevant studies that met all inclusion requirements. From these selected studies, 232 experimental data points were systematically extracted through manual data collection from tables, figures, and text, with each data point cross-verified against original sources to ensure accuracy. The extracted dataset encompasses material compositions (polymer types, concentrations), electrolyte formulations (ionic species, mass fractions), operating conditions (current density, voltage windows, cycle numbers), and electrochemical performance metrics (specific capacitance, cycle stability).

Rigorous data preprocessing procedures were implemented including: (1) removal of duplicate entries from identical experimental conditions, (2) treatment of missing values through literature cross-referencing, (3) elimination of statistical outliers using the interquartile range method (IQR > 1.5), and (4) min-max scaling of continuous variables to normalize value ranges. Categorical features including polymer types and electrolyte systems were systematically encoded using one-hot encoding to create binary numerical representations suitable for machine learning processing.

Polymer types were classified into six categories: Synthetic Vinyl (SV) including PVA, PAM, and PAN; Natural Polysaccharide (NP) encompassing SA, cellulose, chitosan, alginate, and starch; Conductive Polymer (CP) comprising PANI, PPY, PEDOT, and PTH; Polybase (PB), Polyacid (PA), and protein-based materials. Electrolyte systems were categorized as Alkali Metal (AM), Acid System (AS), Zinc System (ZS), Mixed System (MS), and Organic Additive (OA) based on chemical composition.

One-hot encoding was applied to categorical features to create binary numerical representations. The final dataset incorporated 16 input features: PC, AM, AS, ZS, MS, OA, MF, IC, CD, VW, CN, SV, NP, CP, PB, and PA, with specific capacitance and cycle stability as target variables.

### 4.2. ML Model Development

The dataset was split into training and testing sets using an 80:20 ratio to evaluate model generalization performance. Six machine learning algorithms were evaluated: AdaBoost (ADB), Gradient Boosting Regression (GBR), K-Nearest Neighbors (KNN), Support Vector Machine (SVM), XGBoost (version 2.0.2), and Lasso Regression. Tree-based ensemble models were emphasized for their effectiveness in handling categorical features and capturing complex feature interactions in material property prediction.

After model training, validation was performed using the test set, comparing predicted results with actual outcomes to verify the accuracy of different models. Model predictive performance was quantified using key statistical parameters, including root mean squared error (RMSE), mean absolute error (MAE), and coefficient of determination (R^2^). RMSE measures the deviation between predicted and actual values, reflecting prediction accuracy. MAE serves as an intuitive indicator of model error, with values approaching zero indicating higher accuracy. R^2^ indicates the degree of fit between model estimates and observed values, with values approaching 1 demonstrating superior performance. The RMSE, MAE, and R^2^ were calculated using Equations (1)–(3):

(1) $$RMSE=\sqrt{\frac{\sum_{i=1}^{n}{\left(y_{t}-y_{p}\right)}^{2}}{n}}$$

(2) $$MAE=\frac{\sum_{i=1}^{n}\left|y_{t}-y_{p}\right|}{n}$$

(3) $$R^{2}=1-\frac{\sum_{i=1}^{n}{\left(y_{p}-y_{t}\right)}^{2}}{\sum_{i=1}^{n}{\left(y_{t}-y_{m}\right)}^{2}}$$

where yp represents the predicted output value, yt denotes the reported true output value, ym represents the mean of observed output values, n indicates the number of samples in the training or testing datasets. Hyperparameter optimization was conducted using Optuna for XGBoost and grid search for GBR to address overfitting and improve generalization capability. SHAP analysis was implemented to provide model interpretability, quantifying feature contributions and identifying parameter interactions affecting supercapacitor performance.

### 4.3. Model Optimization and Interpretation

Hyperparameter optimization was conducted for the most promising algorithms based on preliminary evaluation. XGBoost was optimized for specific capacitance prediction using Optuna framework, exploring learning rate (0.01–0.3), maximum depth (3–10), number of estimators (50–500), subsample ratio (0.6–1.0), and regularization parameters with 100 trials using Tree-structured Parzen Estimator algorithm.

GBR was optimized for cycle stability prediction using grid search, systematically evaluating number of estimators (50–300), learning rate (0.05–0.2), maximum depth (3–8), and minimum samples split (2–10) to identify optimal parameter combinations.

Model interpretability was achieved through SHAP analysis, providing feature importance ranking, summary plots visualizing feature impact magnitude and direction, and interaction analysis revealing interdependencies between input features. SHAP values quantify the marginal contribution of each feature to individual predictions, enabling mechanistic understanding of relationships between material parameters and electrochemical performance for rational design guidance.

## Data Availability

The data presented in this study are available on request from the corresponding authors.

## Supplementary Materials

The following supporting information can be downloaded at: https://www.mdpi.com/article/10.3390/gels11060464/s1, Figure S1. (a) specific capacitance and (b) cycle stability prediction. Figure S2. Violin plots of prediction error distributions from different ML models for (a) specific capacitance and (b) cycle stability. Figure S3. XGBoost optimization for cycle stability prediction. Figure S4. GBR optimization for specific capacitance prediction.
