# Supplementary material for: Interpretable Machine Learning Analysis of Design Factors in Hydrogel Supercapacitors
Source: Gels. 2025 Jun 18;11(6):464. doi: 10.3390/gels11060464 (PMC12192259; doi:10.3390/gels11060464)
Supplement: Supplementary file 1 [file gels-11-00464-s001.zip › gels-3705894-supplementary.pdf]

Supporting Information

Interpretable Machine Learning Analysis of Design Factors in Hydrogel Supercapacitors

1. Taylor diagrams comparing ML model performance

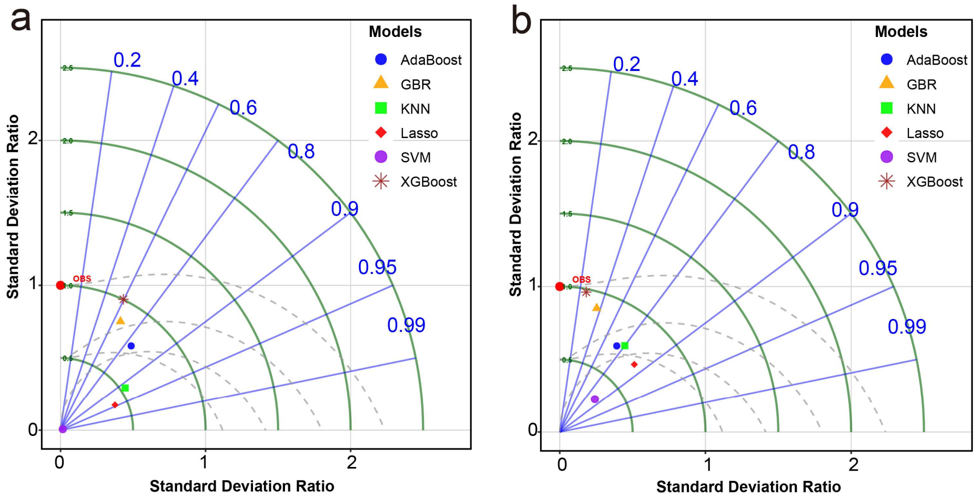

Figure S1. (a) specific capacitance and (b) cycle stability prediction

2. Violin plots of prediction error distributions from different ML models

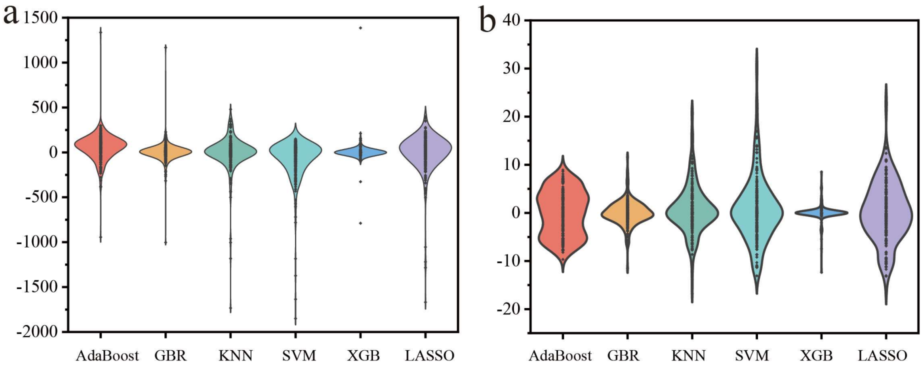

Figure S2. Violin plots of prediction error distributions from different ML models for (a) specific capacitance and (b) cycle stability

3. XGBoost optimization for cycle stability prediction

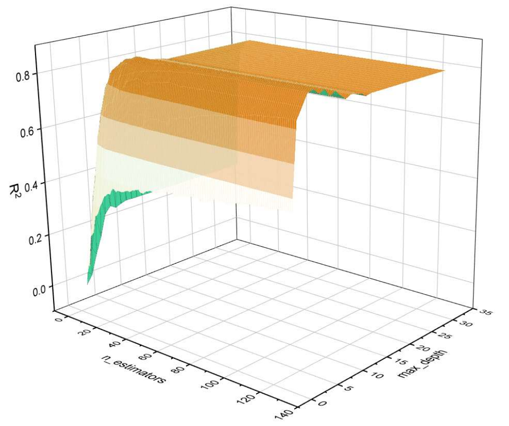

Figure S3. XGBoost optimization for cycle stability prediction

4. GBR optimization for specific capacitance prediction

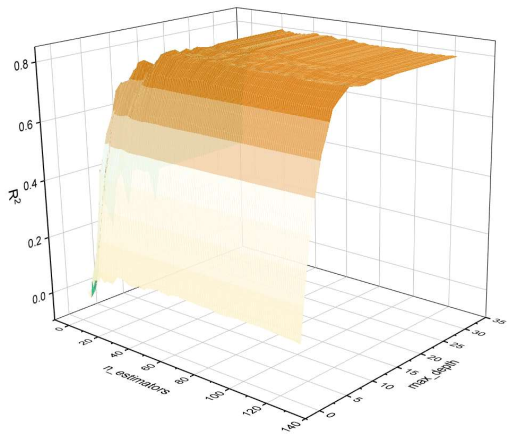

Figure S4. GBR optimization for specific capacitance prediction
